# Comparison between percutaneous transthoracic co-axial needle CT-guided biopsy and transbronchial lung biopsy for the diagnosis of persistent pulmonary consolidation

**DOI:** 10.1186/s13244-023-01436-3

**Published:** 2023-05-11

**Authors:** Juan Wang, Tongyin Zhang, Yanyan Xu, Meng Yang, Zhenguo Huang, Jie Lin, Sheng Xie, Hongliang Sun

**Affiliations:** 1 Department of Radiology, China-Japan Friendship Hospital, No.2 Yinghua East Street, Chaoyang District, Beijing, 100029 China; 2 Graduate School, Chinese Academy of Medical Science & Peking Union Medical College, Beijing, China; 3 Department of Pulmonary and Critical Care Medicine, China-Japan Friendship Hospital, Beijing, China; 4 Department of Pathology, China-Japan Friendship Hospital, Beijing, China

**Keywords:** Pulmonary consolidation, Percutaneous transthoracic CT-guided co-axial needle biopsy, Transbronchial lung biopsy

## Abstract

**Background:**

Diagnosing persistent pulmonary consolidation still faces challenges. The purpose of this study is to compare the diagnostic yield and the complication rate between percutaneous transthoracic CT-guided coaxial needle biopsy (PTCNB) and transbronchial lung biopsy (TBLB) of persistent pulmonary consolidation.

**Materials:**

From January 1, 2016, to December 31, 2020, we have retrospectively enrolled a total of 155 consecutive patients (95 males, 60 females) with persistent pulmonary consolidation who underwent both TBLB and PTCNB. According to the standard reference, the diagnostic yield, accuracy, sensitivity and specificity of PTCNB and TBLB were assessed and compared.

**Results:**

According to the standard reference, the final biopsy diagnoses of 11 cases were confirmed true malignant based on the surgical resections, the remaining were confirmed by clinical and imaging follow-up for at least 12 months. The overall diagnostic accuracy, sensitivity and specificity of PTCNB for malignant diagnosis were 91.61%, 72.34% and 100%, whereas of TBLB were 87.74%, 59.57% and 100%. The diagnostic yield of PTCNB and TBLB were 50.32% and 25.16%, respectively. For the TBLB-based negative cases, PTCNB provided a definite diagnostic yield of 37.93%. There were 45 (29.03%), 22 (14.19%) and 13 (8.39%) patients who experienced pneumothorax, intrapulmonary hemorrhage and hemoptysis, respectively, in PTCNB, while there were only 5 (3.22%) cases of mild intraprocedural bleeding occurring in TBLB.

**Conclusions:**

CT-guided co-axial needle biopsy is an effective and safe modality, associated with higher diagnostic yield and better diagnostic accuracy compared to transbronchial lung biopsy for malignancy presenting as persistent consolidation, especially as the complementary method for TBLB-based negative lung lesions.

**Key Points:**

- Both PTCNB and TBLB showed high diagnostic accuracy for malignancy.
- PTCNB had a higher diagnostic yield than TBLB for persistent pulmonary consolidation.
- PTCNB could provide a complementary diagnosis for TBLB-based negative lung consolidation.

## Introduction

Pulmonary consolidation is a common clinical entity that refers to a group of pulmonary abnormalities, which appears as a homogenous increment in lung parenchymal attenuation that obscures the margins of vessels and airways walls on CT scan [1]. It is mostly due to an acute pulmonary infection. However, if the pulmonary consolidation has failed to resolve by 50% in 2 weeks or completely in 4 weeks, it is considered to be persistent [2–4]. Therefore, it’s important to differentiate malignant from non-malignant causes such as inadequately treated infection, atypical infection, organizing pneumonia, chronic eosinophilic pneumonia, sarcoidosis or vasculitis.

Thus, establishing a histopathological diagnosis seems to be necessary. There are multiple approaches which include sputum cytology, bronchoscopic sampling and percutaneous transthoracic CT-guided co-axial needle biopsy (PTCNB) [5]. Transbronchial lung biopsy (TBLB) with bronchoalveolar lavage (BAL) is the preferred approach theoretically in view of its reduced risk of pneumothorax as it does not traverse the pleural layers [6]. Yet, the diagnostic yield of transbronchial biopsy is low because of many factors, such as peripherally distributed lesions, heterogeneity within a lesion or tissue sample size. Percutaneous transthoracic CT-guided co-axial needle biopsy is another effective strategy for determining diagnosis in persistent pulmonary consolidation, which owns a high diagnostic yield. However, the complications of this procedure are not rare, which include pneumothorax, hemoptysis and hemorrhage [7].

The primary objective of this retrospective study is to compare the diagnostic yield and complication rates of pulmonary persistent consolidation between transbronchial lung biopsy (TBLB) and percutaneous transthoracic CT-guided co-axial needle biopsy (PTCNB) on the same sample.

## Materials and methods

The institutional ethical review board of our hospital has approved this retrospective study and waived the requirement for informed consent for collecting data from the related patients. The written informed consents for CT-guided coaxial core needle biopsy and transbronchial biopsy had been obtained from all patients prior to performing the procedures.

### Study subjects

All patients who accepted both CT-guided percutaneous transthoracic co-axial needle biopsy and transbronchial biopsy were identified by picture archiving and communication system (PACS) and electronic medical records. From January 2016 to December 2020, a total of 2363 patients underwent intervention procedures for persistent pulmonary consolidations. The persistent consolidation was defined as an opacity that obscures the margins of vessels and/or airway walls and failed to resolve 50% in 2 weeks or completely in 4 weeks [2–4]. The exclusion criteria were as follows: (1) A consolidation presenting as obstructive opacity by a mass or nodule on CT [1]; (2) Patients who only received CT-guided biopsy or TBLB; (3) No standard reference for inclusive biopsy (i.e., no postoperative histopathological confirmation or no 12-month clinical follow up). Totally, 155 patients subjected to both CT-guided lung biopsy and transbronchial examination within 1 week were included in this study. Since TBLB examination obtains tissue sample from natural airway without radiation exposure and can be performed by a physician, TBLB is the priority in clinical routine items. Of all patients, 120 underwent TBLB examination before PTCNB, and the remaining did TBLB examination after PTCNB.

Before the biopsy (either CT-guided biopsy or bronchoscopic biopsy), coagulation profile, platelet counts and prothrombin factors were ordered routinely. Antiplatelets or anticoagulant agents (e.g., aspirin, clopidogrel, warfarin, etc.) had been discontinued 7 days prior to biopsy. Furthermore, the clinical data and demographic information were obtained from the electronic medical records, including smoking history, past cancer history, past thoracic operative history and previous PET/CT examinations. If a patient received a PET/CT examination within 30 days, percutaneous biopsy could be guided based on the hypermetabolic region in the previous PET/CT scan (43/155; 27.74%).

### PTCNB procedure

The patient was positioned in the supine, prone or lateral position, depending on the location of the target lesion. Before the biopsy, the patients were instructed to breathe slowly and then hold the breath at the end of inspiration.

A 16-detector-row scanner (Aquilion 16; Canon Medical Systems) was used; the relative parameters were: (1) scanning method = helical acquisition mode; (2) tube currents = automatic tube current modulation; (3) tube voltage = 120 kVp; (4) slice thickness = 4–5 mm. Only the lesion area was scanned in order to reduce the radiation dose. Then, the operators chose the appropriate approach to the lesion according to these images, with some extra considerations taken into account, which include: (1) attention to avoid the bone tissues; (2) avoiding the heart and the large blood vessels; (3) avoiding pulmonary bullae and emphysematous region; (4) perpendicular to the pleura as much as possible; (5) the distance of traversing the lung tissues should be as short as possible; (6) away from the interlobar fissure as much as possible [8].

**Fig. 1:**
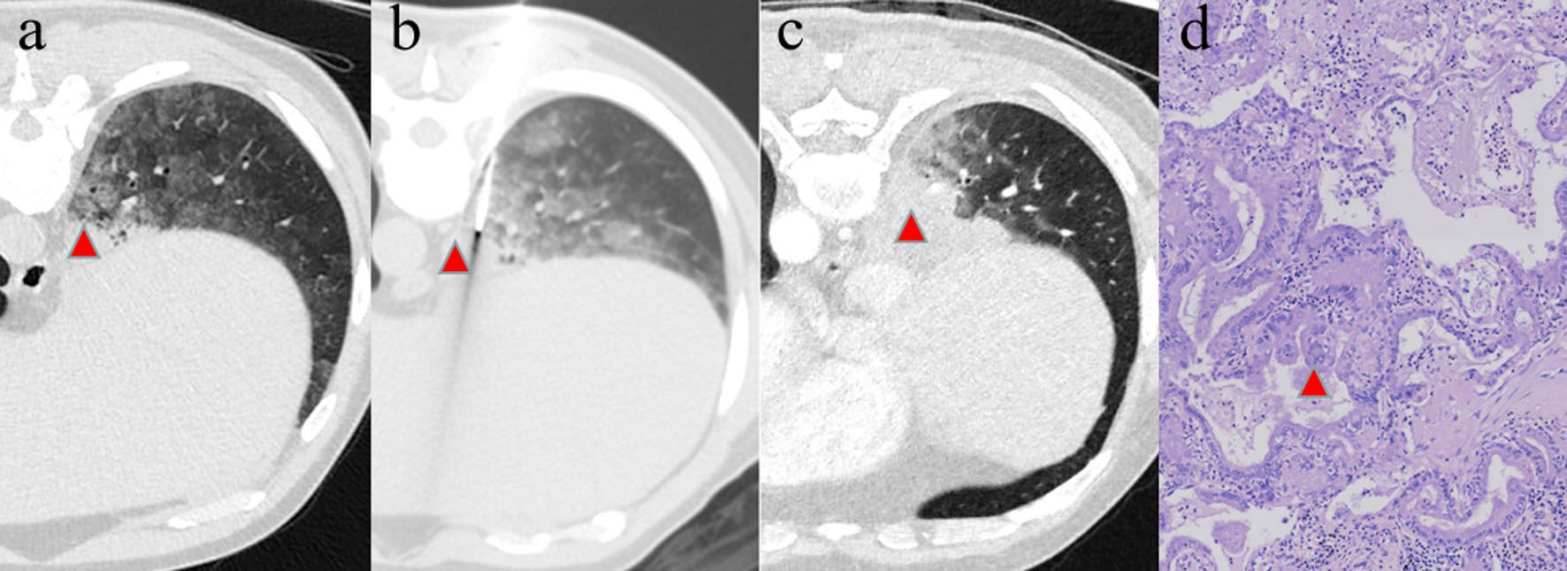
A 57-year-old male non-smoker with persistent consolidation (**a**–**c**, red triangle) surrounded by ground glass opacity in the right lower lobe for 5 months. Anti-infection treatment was ineffective. TBLB was performed first, however, there was nothing remarkable in the biopsy result. **b** CT-guided lung biopsy was performed with patient in prone position, which revealed lung adenocarcinoma. **c** Axial CT scan suggested the area of consolidation progressively expanded in two months. **d** He later underwent surgical resection, and the microscopic examination (hematoxylin–eosin staining, × 200) demonstrated invasive mucinous adenocarcinoma

**Fig. 2:**
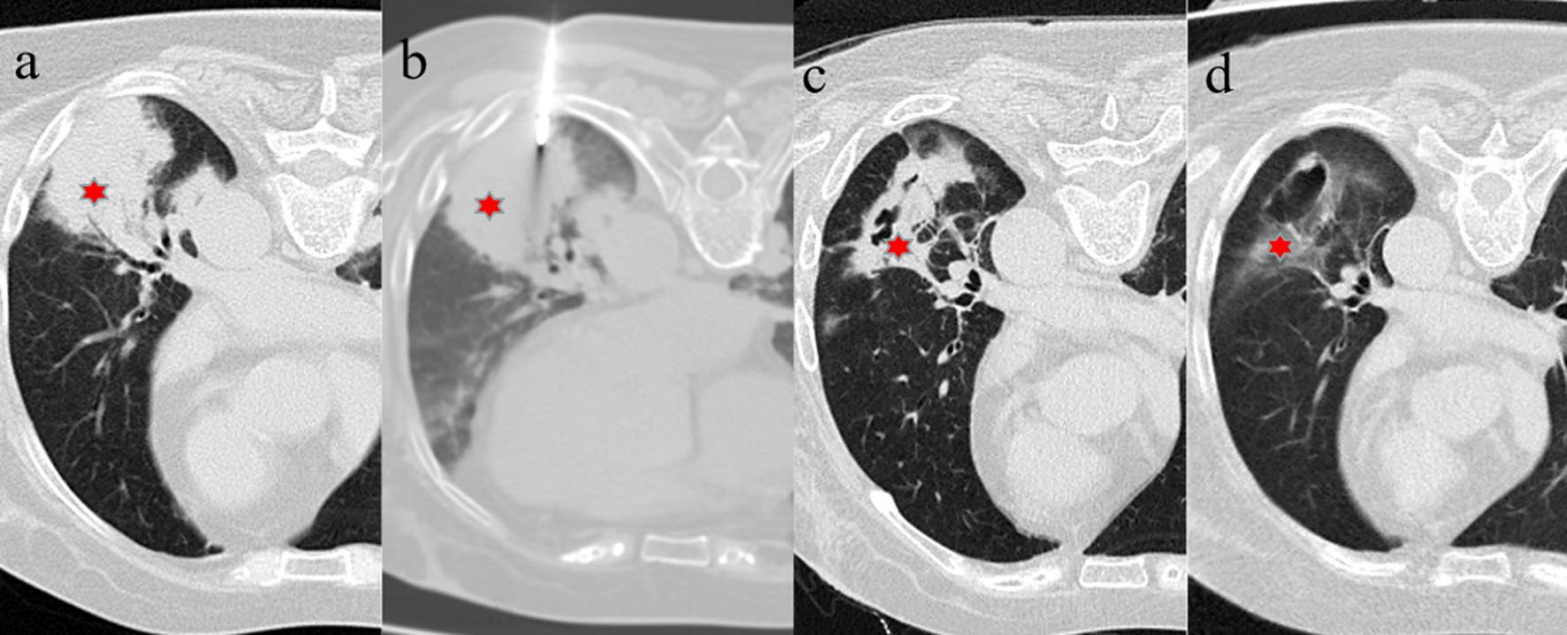
A 66-year-old female presented with a large area of consolidation in the left lower lobe (**a**, red star). An empiric antibiotic therapy was ineffective. TBLB carried out prior to PTCNB revealed a non-specific infection. PTCNB then indicated a cryptococcus infection. **b** An Axial CT scan was obtained during PTCNB. **c** and **d** were the follow-up CT scans performed 6 months and 18 months, respectively, after biopsy. A gradual absorption process was observed after undergoing anti-fungal treatment, which confirmed the biopsy result

In terms of the above principles, an appropriate puncture point on a patient’s skin was marked. Subsequently, the skin of the puncture area was disinfected and 1% lidocaine (5 mL) was administered as a local anesthesia. Then a 10-cm-long 17/18-gauge percutaneous introducer needle (Cook Incorporated) was advanced to puncture from the marked point on the skin without penetrating the parietal pleura. A local CT scan with a 1.6 cm range centered on the needle insertion plane was performed to confirm the direction of the needle’s tip. After the needle was further advanced into the target lesion, the third CT scan was carried out to verify the final position of the needle tip. 2–3 specimens with a length of 1 cm were then obtained with BioPince™ full core biopsy instrument (Argon Medical Devices, Inc.) and sent for histopathology diagnosis examination (Figs. 1 and 2).

Eventually, a post-biopsy CT scan of the lesion area was performed to rule out the occurrence of the possible complications such as pneumothorax, intrapulmonary hemorrhage around the lesion and the puncture needle tract, hemothorax, air embolism and so on. The severity of the related complications was evaluated according to the Society of Interventional Radiology Standards of Practice Committee classification of complications [9].

### TBLB procedure

Bronchoscopic biopsy for persistent pulmonary consolidation can be performed by using several different instruments and sampling methods, such as transbronchial biopsy forceps, transbronchial brush, transbronchial needle aspiration, radial probe endobronchial ultrasound-guided transbronchial lung biopsy (EBUS-TBLB) and electromagnetic navigational bronchoscopic biopsy (ENB) [5]. However, we consider them as TBLB in this study.

Before bronchoscopy, patients were requested to fast for 6 h. Conscious sedation was subsequently achieved. The choice of anesthesia was left at the discretion of the anesthetist. The bronchoscope was passed through the nose or the mouth, and then through the vocal cords into the trachea. The physician completed endobronchial inspection of all segments of bilateral lungs to exclude significant endobronchial abnormalities. Under either imaging or fluoroscopic guidance, the patient was asked to hold their breath, then 5–12 specimens were obtained by a forceps. Some samples were sent for histopathology examination, others for pathogenic analysis. Besides that, a bronchoalveolar lavage was performed to obtain specimens for culture and cytology tests prior to the TBLB. After obtaining the samples, the physician retrieved the bronchoscope after noting that there was no active bleeding.

### Biopsy results and final diagnosis

According to the histopathological and microbiological analyses, all biopsy results of PTCNB/TBLB were divided into four categories: (1) malignant or suspicious for malignancy (the specimens showed malignant cells or findings suspicious for malignancy); (2) specific benign (the results revealed benign tumors, a special infection, or a non-neoplastic condition such as hamartoma, aspergillus, vasculitis); (3) non-specific benign (the results demonstrated fibrosis, necrosis or inflammation without identification of a specific disease); (4) non-diagnostic (the specimens contained only lung parenchyma, respiratory epithelial cells, histiocytes or blood). The final diagnosis was determined by surgical biopsy and at least 12 months of clinical and imaging follow-up findings.

The malignant or suspicious of malignancy specimen was considered to be a positive result and the others (specific benign, non-specific benign and non-diagnostic) were considered to be negative results. If a positive biopsy result correlated with the final diagnosis, it was considered to be a true positive; in contrast, it was a false positive. Similarly, if a negative biopsy result was consistent with the final diagnosis, it was considered to be a true negative, and if not, it was a false negative.

According to the above definition, the diagnostic yield of PTCNB/TBLB in the evaluation of persistent lung consolidation was defined as the percentage of biopsy result demonstrating as a true diagnosis of malignant or suspicious of malignancy or specific benign. The diagnostic accuracy, sensitivity, specificity, positive and negative predictive values for malignancy between PTCNB and TBLB were calculated and compared.

### Statistical analysis

Statistical analysis was completed using SPSS software (SPSS 26.0 for Windows, SPSS, Chicago, IL). Statistical difference in continuous variables was shown as mean ± SD. Risk factors of diagnostic failure in TBLB-based negative cases were identified using χ^2^ test or Fisher exact probability tests. *p* ≤ 0.05 was considered to indicate a statistical significant difference.

## Results

The technical success rate of CT-guided biopsy and transbronchial biopsy was 100%. From January 1, 2016, to December 31, 2020, there was a total of 155 subjects (95 males, 60 females, with a mean age of 58.83 ± 15.25 (SD) years; ranging from 15 to 84 years of age) who underwent both CT-guided percutaneous biopsy and transbronchial biopsy that were enrolled in this study. Only 6 patients had a previous cancer history and 3 of them received thoracic operations previously. For all patients, 11 (11/155; 7.10%) received surgery, and 144 (144/155; 92.90%) were determined by clinical and imaging follow-up findings. Basic clinical characteristics of the patients are summarized in Table 1.

**Table 1:** Summary of basic clinical characteristics for 155 patients

Variable		Value
Age (year)	Mean ± SD (range)	58.83 ± 15.25 (15–84)
Gender	Male/female	95/60
Smoking status	Former/current/never	41/22/92
Cancer history	Pulmonary/extra-pulmonary/no	1/5/149
Previous thoracic operation	Yes/no	3/152
Use of anticoagulants	Yes/no	19/136
PET/CT prior to biopsy	Yes/no	43/112
Biopsy site	Peripheral/central	139/16
Standard reference for biopsy diagnosis	Surgery/follow-up 12-month	11/144
Final diagnosis	Malignant/Benign/Non-specific benign	47/68/40
Procedure-related complications	Yes/no	62/93
	Pneumothorax/intrapulmonary hemorrhage/hemoptysis/others	45/22/13/2

SD, standard deviation; PTCNB, percutaneous transthoracic needle biopsy; TBLB, transbronchial lung biopsy; PET/CT, positron emission tomography/computed tomography

**Fig. 3:**
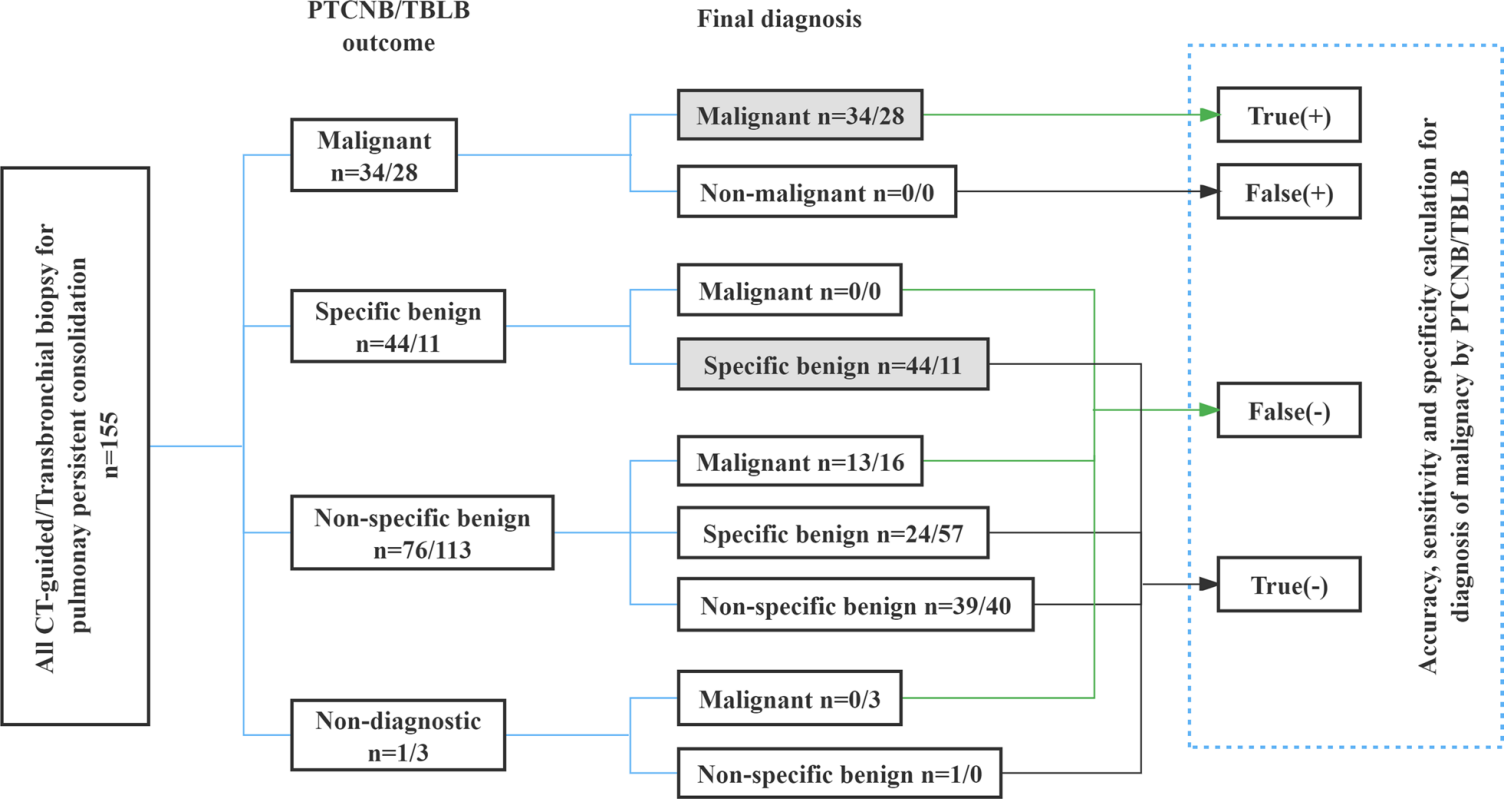
A flowchart demonstrating PTCNB/TBLB outcome and the final diagnosis of the subjects are included, as well as the calculations for accuracy, sensitivity, specificity of PTCNB/TBLB for malignancy. In addition, the cases in gray boxes represent biopsy success group in this study

We performed the two biopsy procedures on the same sample. PTCNB/TBLB outcomes and the final diagnoses are shown in Table 2. According to the standard reference, the final diagnosis includes 47 malignant lesions, 68 specific benign lesions and 40 non-specific benign lesions. Of them, 11 cases were proved to be malignant by surgical resection, whereas the remaining 36 cases were confirmed to be malignant when the lesion size was reduced or enlarged after radiotherapy, chemotherapy, or targeted therapy. All the benign lesions were followed up for at least 12 months either clinically or with imaging by at least 12-month clinical and imaging following findings. The overall diagnostic accuracy, sensitivity and specificity of PTCNB for malignant diagnosis were 91.61% [(34 + 108)/155], 72.34% [34/(34 + 13)], 100% [108/(108 + 0)], and of TBLB were 87.74% [(28 + 113)/155], 59.57% [28/(28 + 19)], 100% [113/(113 + 0)]. The overall diagnostic accuracy, sensitivity and specificity of PTCNB for benign diagnosis were 84.52% [(44 + 87)/155], 64.71% [44/(44 + 22)], 100% [87/(87 + 0)], and of TBLB were 63.23% [(11 + 87)/155], 16.18% [11/(11 + 57)], 100% [87/(87 + 0)]. The diagnostic yield between PTCNB and TBLB were 50.32% [(34 + 44)/155] and 25.16% [(28 + 11)/155], respectively (Fig. 3). In conclusion, the whole diagnostic yield and diagnostic accuracy rate of PTCNB were higher than that of TBLB.

**Table 2:** Outcome and final diagnosis of PTCNB/TBLB in 155 patients

Lung lesion	TBLB outcome	PTCNB outcome	Final diagnosis
**Malignant**	**28 (28/155; 18.06%)**	**34 (34/155; 21.94%)**	**47 (47/155; 30.32%)**
Lung adenocarcinoma	15 (15/155; 9.68%)	26 (26/155; 16.77%)	29 (29/155; 18.71%)
Lung squamous carcinoma	8 (8/155; 5.16%)	5 (5/155; 3.23%)	12 (12/155; 7.74%)
Sarcomatoid carcinoma	1 (1/155; 0.65%)	0 (0/155; 0.00%)	1 (1/155; 0.65%)
Neuroendocrine carcinoma	3 (3/155; 1.94%)	2 (2/155; 1.29%)	3 (3/155; 1.94%)
Adenosquamous carcinoma	1 (1/155; 0.65%)	1 (1/155; 0.65%)	1 (1/155; 0.65%)
Lymphoma	0 (0/155; 0.00%)	0 (0/155; 0.00%)	1 (1/155; 0.65%)
**Specific-benign**	**11 (11/155; 7.10%)**	**44 (44/155; 28.39%)**	**68 (68/155; 43.87%)**
Tuberculosis	7 (7/155; 4.52%)	14 (14/155; 9.03%)	15 (15/155; 9.68%)
Organized pneumonia	1 (1/155; 0.65%)	24 (24/155; 15.48%)	26 (26/155; 16.77%)
Sarcoidosis	2 (2/155; 1.29%)	2 (2/155; 1.29%)	3 (3/155; 1.94%)
Abscess	0 (0/155; 0.00%)	1 (1/155; 0.65%)	13 (13/155; 8.39%)
Cryptococcus	0 (0/155; 0.00%)	1 (1/155; 0.65%)	1 (1/155; 0.65%)
Pneumocystis carinii pneumonia	1 (1/155; 0.65%)	1 (1/155; 0.65%)	1 (1/155; 0.65%)
Actinomycetes	0 (0/155; 0.00%)	0 (0/155; 0.00%)	1 (1/155; 0.65%)
Non-tuberculous mycobacteria	0 (0/155; 0.00%)	0 (0/155; 0.00%)	1 (1/155; 0.65%)
Benign tumor	0 (0/155; 0.00%)	1 (1/155; 0.65%)	2 (2/155; 1.29%)
Vasculitis	0 (0/155; 0.00%)	0 (0/155; 0.00%)	3 (3/155; 1.94%)
Sicca syndrome related disease	0 (0/155; 0.00%)	0 (0/155; 0.00%)	1 (1/155; 0.65%)
Non-tuberculous mycobacteria	0 (0/155; 0.00%)	0 (0/155; 0.00%)	1 (1/155; 0.65%)
**Non-specific benign**	**113 (113/155; 72.90%)**	**76 (76/155; 49.03%)**	**40 (40/155; 25.81%)**
**Non-diagnostic**	**3 (3/155; 1.94%)**	**1 (1/155; 0.65%)**	**0 (0/155; 0.00%)**

Among the 116 cases of TBLB-based diagnostic failure, CT-guided core needle biopsy yielded a definite diagnosis for 44 lesions (37.93%), which included 14 malignant and 30 specific benign lesions. The remaining 72 PTCNB-based negative lesions consisted of 5 malignant, 1 non-diagnostic and 66 non-specific lesions. The overall accuracy, sensitivity and specificity of PTCNB for malignancy in TBLB-based negative lesions were 95.69% [(14 + 97)/116], 73.68% [14/(14 + 5)], and 100% [97 + 0)/97], respectively. Table 3 provides the results to analyze the risk factors for diagnostic failure. It is demonstrated that there is a significant difference between the two groups in diagnosing specific benign lesions (*p* < 0.001).

**Table 3:** Basic data of TBLB-based negative lesions in 116 patients

TBLB-based negative lesions		PTCNB success (n = 44)	PTCNB failure (n = 72)	*P* value
Age	Mean ± SD	58.75 ± 15.36	59.97 ± 14.08	0.363
Gender	Male/female	28/16	39/33	0.316
Smoking status	Ex-or current/never	22/22	24/48	0.075
Specific benign lesions	Yes/no	30/14	27/45	< 0.001
Cancer history	Yes/no	3/41	3/69	0.532
Central lesion	Yes/no	16/28	36/36	0.152
Presence of cavitation	Yes/no	2/42	5/67	0.599
Location	Right/left lung	29/15	40/32	0.270
Diameter	< 3 cm/ ≥ 3 cm	9/35	13/59	0.749
Pneumothorax	Yes/no	16/28	20/52	0.332
Hemoptysis	Yes/no	2/42	9/63	0.156

### Complications

Among 155 CT-guided percutaneous lung biopsies of persistent pulmonary consolidation, the occurrence of pneumothorax, intrapulmonary hemorrhage and hemoptysis are 45 (45/155; 29.03%), 22 (22/155; 14.19%) and 13 (13/155; 8.39%) respectively. However, none of them needed further treatment. Subcutaneous emphysema occurred in one case after CT-guided biopsy. Only one patient had massive hemoptysis after CT-guided biopsy, and subsequently developed dyspnea, then required tracheal intubation and chest compression; finally, the patient was discharged with an improved general condition. There were less complications during transbronchial biopsy procedures, which only include 5 (5/155, 3.22%) cases of intraprocedural bleeding in our study.

## Discussion

To our knowledge, the studies comparing CT-guided lung biopsy and transbronchial lung biopsy on persistent consolidation were limited [5, 10–13]. Therefore, there were no differences in variables except biopsy methods between the two groups. Both of the procedures showed high diagnostic accuracy (91.61% vs 87.74%), sensitivity (72.34% vs 59.57%) and specificity (100.00% vs 100.00%) for malignancy presenting as a persistent consolidation on CT scan in our study. However, similar to data reported by other investigators, our study also revealed a significantly higher diagnostic yield for CT-guided approach compared to bronchoscopic sampling (50.32% vs 25.16%). The reason may be that TBLB is limited in its ability to obtain adequate samples [14], whereas CT guidance can ensure that the needle enters into the lesion when a biopsy is taken [15, 16]. Besides, some studies revealed that the success of transbronchial examination depends on the location of the lesions, where the sensitivity of bronchoscopic procedure for central lesions is higher than that for peripheral lesions [17]. However, there was no significant difference in our study, which can be explained by the reason that the size of the lesions in our study were mostly ≥ 3 cm (128; 82.58%), which has involved both central and peripheral lung parenchyma. Nevertheless, compared with the previous studies about nodules and masses biopsy, both PTCNB and TBLB had a lower diagnostic yield of 50.32% and 25.16%, respectively, for pulmonary consolidation, which was possibly because the consolidations may contain other components such as obstructive lesions, necrosis, fibrosis and so forth, thus affecting the selection of puncture target. PET/CT fusion imaging might help differentiate tumor and other tissues, in view of as it allows us to obtain a specific tissue sample for a clear pathological verification by targeting the hypermetabolic area among the consolidation. Our previous study revealed that a prior PET/CT fusion imaging could improve the diagnostic yield of CT-guided transthoracic core-needle biopsy [18]. On the other hand, when multiple consolidations occurred simultaneously in a patient, only the outcome of the biopsied lesions were analyzed, which might decrease the diagnostic accuracy. Furthermore, there were some biopsied samples of benign lesions containing only chronic inflammatory cells, such as lymphocyte or plasmacyte, and some fibrous tissues. Subsequently, these cases were confirmed to be non-specific benign by clinical and imaging follow-up and finally classified to diagnostic failure in our study.

As for complications, there was certainly a higher incidence in PTCNB compared to TBLB. Nonetheless, most of them were self-limiting and within an acceptable range compared to its considerable diagnostic yield. In addition, we found the diagnostic rate of CT-guided lung biopsy was also considerably high (37.93%) in patients with TBLB-based diagnostic failure in our study. Furthermore, there was also a significant difference in diagnosing specific benign lesions between PTCNB and TBLB (*p* < 0.001). Considering these findings, CT-guided biopsy may complement transbronchial examination for patients with TBLB-based negative lung consolidation [17, 19].

There are several limitations in the current study. Firstly, given the retrospective study design, only patients who underwent both PTCNB and TBLB within 1 week were enrolled in this study. However, our study is the cohort performed in patients with pulmonary consolidation, which compares the two methods in the same patient. Next, the biopsy site of the two methods were possibly not identical, which may result in a discrepancy between the two methods. Then, the imbalanced sample size between the CT-guided biopsy and TBLB also caused the selection bias. Furthermore, there is no consensus on the duration of follow-up, a 12-month follow-up which is similar to other studies may not have been sufficient. Finally, the outcome of CT-guided biopsy and TBLB might be closely related to the operators’ experience [20], and the result in our study was the summary for the procedures performed by two experienced interventional radiologists and one clinical respiratory physician, respectively, in a single medical center.

In conclusion, CT-guided coaxial needle biopsy is an effective and safe modality, associated with higher diagnostic yield and better diagnostic accuracy compared to transbronchial lung biopsy for malignancy, which presents as a persistent consolidation, especially when performed as a complementary method for TBLB-based negative lung lesions.

## Data Availability

The datasets used and/or analyzed during the current study are available from the corresponding author on reasonable request.

## Abbreviations

BAL: Bronchoalveolar lavage
PACS: Picture archiving and communication system
PET/CT: Positron emission tomography/ computed tomography
PTCNB: Percutaneous transthoracic CT-guided coaxial core needle biopsy
SD: Standard deviation
TBLB: Transbronchial lung biopsy
